# A novel hybrid bioprocess strategy addressing key challenges of advanced biomanufacturing

**DOI:** 10.3389/fbioe.2023.1211410

**Published:** 2023-06-30

**Authors:** Lucas Nik Reger, Martin Saballus, Annika Kappes, Markus Kampmann, Rene H. Wijffels, Dirk E. Martens, Julia Niemann

**Affiliations:** ^1^ Corporate Research, Sartorius, Göttingen, Germany; ^2^ Bioprocess Engineering, Wageningen University, Wageningen, Netherlands

**Keywords:** CHO cell culture, process intensification, fluidized bed centrifuge, monoclonal antibodies, intermediate harvest, continuous biomanufacturing

## Abstract

Monoclonal antibodies (mAb) are commonly manufactured by either discontinuous operations like fed-batch (FB) or continuous processes such as steady-state perfusion. Both process types comprise opposing advantages and disadvantages in areas such as plant utilization, feasible cell densities, media consumption and process monitoring effort. In this study, we show feasibility of a promising novel hybrid process strategy that combines beneficial attributes of both process formats. In detail, our strategy comprises a short duration FB, followed by a fast media exchange and cell density readjustment, marking the start of the next FB cycle. Utilizing a small-scale screening tool, we were able to identify beneficial process parameters, including FB interval duration and reinoculation cell density, that allow for multiple cycles of the outlined process in a reproducible manner. In addition, we could demonstrate scalability of the process to a 5L benchtop system, using a fluidized-bed centrifuge as scalable media exchange system. The novel process showed increased productivity (+217%) as well as longer cultivation duration, in comparison to a standard FB with a significantly lower media consumption per produced product (−50%) and a decreased need for process monitoring, in comparison to a perfusion cultivation. Further, the process revealed constant glycosylation pattern in comparison to the perfusion cultivation and has strong potential for further scale-up, due to the use of fully scalable cultivation and media exchange platforms. In summary, we have developed a novel hybrid process strategy that tackles the key challenges of current biomanufacturing of either low productivity or high media consumption, representing a new and innovative approach for future process intensification efforts.

## 1 Introduction

The development and manufacturing of biopharmaceuticals including monoclonal antibodies (mAb) have gained major importance due to their growing demand in treatments of cancerous, immunological and infectious diseases (Johnson, 2018). MAbs offer higher target specificities and elongated half-lives compared to pharmaceuticals of low molecular weight, such as small molecule drugs, resulting in a high interest in these molecules as demonstrated by increasing numbers of antibodies entering clinical trials, and getting regulatory approval (Hansel et al., 2010). Additionally, their mode of action can be modified and optimized in various ways by molecular engineering (Presta, 2008). However, to achieve the desired bioactivity and avoid immunogenicity, host-specific posttranslational modifications (PTM) of the expressed mAb, like glycosylation, are essential (Jayapal et al., 2007). Therefore, mAbs are commonly expressed in mammalian cells capable of PTM, especially in Chinese Hamster Ovary (CHO) cells due to their status as secure production host and their proven high volumetric productivity (Kim et al., 2012). Thereby, different process modes are utilized within the upstream manufacturing of mAb based therapeutics comprising distinct sets of advantages and disadvantages.

A commonly used upstream process operation in mAb production is the fed-batch (FB) mode, comprising feed additions throughout a batch cultivation to supply fresh nutrients to the cells. This process comprises an exponential growth phase in which cell numbers increase and cell viability is high, followed by a stationary- and death phase with stable or decreasing viable cell counts and decreasing viability. Commonly, the accumulation of metabolic byproducts or nutrient limitation triggers the transition from the growth phase to the stationary phase and hence, limits the achievable cell density, cultivation time and volumetric productivity (Abu-Absi et al., 2014). The advantages of the FB operation are efficient utilization of growth media in comparison continuous processes, resulting in lower operational costs which increases the economic potential (Pollock et al., 2013).

To overcome the FB specific limitations, like decreased cell densities and cultivation durations, continuous processes like perfusion are applied (Hiller et al., 2017). Thereby, a continuous media exchange is applied, while cells are kept in the reactor using cell retention devices, which usually are filter-based-systems in either tangential flow or alternative tangential flow. Alternative cell retention can be achieved, for example, by using hydrocyclones, acoustic separation or gravitational settlers (Castilho and Medronho, 2002; Voisard et al., 2003; Bettinardi et al., 2020). Thereby, spent media containing the product, residual nutrients as well as metabolic byproducts passes the cell retention device and consecutive enters the downstream operations, meanwhile new media is added to the process to supply fresh nutrients. With the application of cell bleed, i.e., removing spent medium with cells, a pseudo-steady state can be introduced, allowing for stable high cell densities. This process modification allows for an up to 5-fold increase in volumetric productivity and can strongly enhance plant utilization compared to a fed-batch process (Dowd et al., 2003; Xu et al., 2017). However, high media expenses due to media exchange rates up to 3 bioreactor volumes per day, limited filter capacities and operational complexity are the downsides of perfusion processes (Hiller et al., 2017; MacDonald et al., 2022).

Besides the two main process operations, novel process intensification strategies have emerged during recent years, aiming to combine on FB and perfusion cell culture techniques to reach high volumetric productivities as in perfusion with a reduced use of medium as in fed-batch. One example comprises enhanced cell counts in the seed train, facilitated by perfusion operation, followed by a FB operation in the production bioreactor. This intensification strategy not only allows for significantly reduced seed culture volumes and steps as well as increased n-stage inoculation cell densities that consecutively eliminate the less productive initial growth phase in the production reactor (Schulze et al., 2021). This so called high inoculation FB decreases the total process time, while keeping final mAb titers constant, resulting in an overall increase in process productivity (Yang et al., 2014; Yongky et al., 2019; Schulze et al., 2022). Another approach by (Hiller et al., 2017) involves a perfusion process in the beginning of the cultivation to increase the overall cell density, subsequently the process is changed to a FB operation to minimize media consumption. Thereby, the overall output of the process could be enhanced with a relatively low increase of media consumption. A third hybrid approach recently demonstrated by our group is the so-called intermediate harvest (IH) strategy, a single and fast media exchange during a FB operation done by a fluidized-bed-centrifuge (FBC) (Reger et al., 2023). By doing a single media exchange in this way increased peak cell densities as well as volumetric productivities could be achieved similar to the process from (Hiller et al., 2017). All of these intensified processes rely on a beneficial combination of high cell densities of the perfusion culture mode and the low media consumption of the FB process mode, However, all introduced intensification have a limited process duration and thus still result in a discontinuous process mode. Therefore, a process with continuous characteristics alongside to low media consumption and high cell densities is highly desirable.

The aim of this study was to identify a possible process scheme to enable a continuous process operation based on a hybrid process strategy between perfusion and fed-batch. Thereby, this new process should combine increased volumetric productivities, as achieved in common discontinuous FB process, with significant lower media consumption as required in typical continuous perfusion cultivation. In addition, the process should comprise comparably low process control and monitoring needs. To enable this hybrid process strategy the rapid media exchange by Intermediate Harvests (IH) should be utilized, followed by a short duration fed-batch (FB cycle). This process will be further referred to as continuous fed-batch (cFB). In a first step, the feasibility and limitations of this new concept should be investigated in a 15 mL small-scale system utilizing a IH mimic method. Thereby, different re-inoculations densities and FB durations should be evaluated. In a second step the most promising concept should be scaled-up to benchtop-scale to demonstrate feasibility, and further process optimization. Subsequently, a comparison to the standard process, FB and steady-state perfusion, should be conducted.

## 2 Materials and methods

### 2.1 Cell line and medium

A DHFR-deficient Chinese hamster ovary (CHO- DG44) monoclonal cell line was used, stably expressing an IgG_1_ (pI: 7.3) product. The chemically defined 4Cell SmartCHO (Sartorius Stedim Biotech) media system, comprising the Stock and Adaption media as well as the Production Media with a two feed solution (Feed A and Feed B) was used for all n-stage processes as well as seed train cultivations. All seed trains were conducted with similar passage counts to secure consistency.

### 2.2 Process characterization: small scale trials

The small-scale screening was conducted in an Ambr15 (Sartorius Stedim Biotech) high throughput system. Thereby, re-inoculation densities were tested from 20 × 10^6^ to 30 × 10^6^ cells/mL and FB durations between 2 and 3 days. After preparation of the system, temperature setpoint was set to 36.8°C, pH was controlled at 7.1 through CO_2_ gassing. DO was controlled at 40% with O_2_ gassing and stirrer speed set to 1.300 rpm with a liquid volume at around 11 mL. Bioreactors were inoculated with 0.3 × 10^6^ cells/mL. After a 72-h batch phase, daily bolus feeds (Feed A with 4% and Feed B with 0.4%) with decreasing volumetric percentage and glucose (setpoint 4.5 g/L) were started. On day 6, all cultures were processed by using the semi-perfusion method (Janoschek et al., 2019) to mimic the FBC operation and consecutive fresh media enriched basal media according to Janoschek et al. (2019) was supplied to each vessel. Subsequent, a flexible feeding scheme was implemented based on the daily viable cell volume. Thereby, the viable cell volume on peak cell density of the common FB process (34.9 mm^3^/mL) was fixed as single multiplication factor. Subsequent, the viable cell volume was determined daily and the multiplication factor was calculated and applied to the feed. Feeding volumes were split into two boluses for feeding 12 h apart. For all cultures the vessels were changed at day 12. On days of IHs, feeding scheme was adjusted to a single half feed to avoid overfeeding of cells cultured. Cell cultures were ended when reaching 70% cell viability or when observing stagnating cell growth between IHs.

### 2.3 Proof of concept: 5 L benchtop scale

The proof of concept was conducted in a 5 L Univessel (Sartorius Stedim Biotech) system utilizing the 2-day IH interval with re-inoculation at 20 × 10^6^ cells/mL for a total of six repetitions. Two different sets of experiments were done mainly differing in the wash media matrix and re-inoculation matrix for the FBC operations further explained in Section 2.4. Bioreactors were inoculated with 0.3 × 10^6^ cells/mL from seed-cultures with a working volume of 3 L and similar DO, pH and temperature settings. Similar to small scale, an initial batch phase for 72 h was conducted with subsequent daily bolus feeding of the two complementary feeding solutions. On day 5, the cultures were processed by the FBC operations, with a subsequent cell bleed to reach a starting density of 20 × 10^6^ cells/mL. Similar to the small scale a flexible fixed feeding twice a day in a multiplication factor of IVCV was implemented, on harvest days a decreased feeding was utilized to diminish possibility of excessive feeding. After six repetitions the cultivation was ended.

### 2.4 FBC operation

For cell separation, concentration, and washing, an automated single-use centrifuge Ksep^®^ 400 system with a Ksep^®^ 400 Cell Wash Harvest Consumable Kit (Sartorius) was used. Thereby, the FBC operation consists of the steps loading, washing and discharging in cyclic repetition. The tubing of all receptions to the FBC system was sterile connected by a BioWelder^®^ system (Sartorius) and disconnected by a BioSealer^®^ TC system (Sartorius).

To achieve almost complete cell retention and replacement of the spent medium during each IH step by the FBC, cell broth was loaded and washed according to a protocol established by (Saballus et al., 2021). In brief, the FBC was set to constant 1,000 × g at flow rate of 100 mL/min/chamber for loading and washing, with a maximal loading per chamber of around 90%. High concentration of the washed cells was enabled by an adapted FBC discharging recipe of the processed cells, applying an initial dump volume of 100 mL with a subsequent harvest volume of 300 mL at a harvest flow rate of 300 mL/min per FBC chamber. Both tested wash media, the pH 7.4 phosphate buffered saline (PBS, chemicals supplied by Carl Roth) and the fresh enriched basal medium generated according to Janoschek et al. (2019) were pre-tempered at 37°C, the subsequent FBC process was not temperature controlled.

### 2.5 Sampling and analytics

Cell characteristics were measured by Cedex HiRes (Roche), metabolic parameters and pH by ABL800 basic (Radiometer) and ammonia analyzed via BioProfile^®^ FLEX2™ (Nova Biomedical). Residual samples were centrifuged at 6600 × g for 5 min (Centrisart A-14, Sartorius) at RT and stored at −20°C until further analysis.

IgG concentrations were determined by size exclusion chromatography (SEC) utilizing the Vanquish™ Flex UHPLC System (Thermo Scientific) and Yarra™ 3 μm SEC-3000 (Phenomenex). For each measurement a standard curve with an IgG specific standard was prepared. The running buffer included 100 mM Na_2_SO_4_, 50 mM NaH_2_PO_4_ and 50 mM Na_2_HPO_4_ with pH 6.6 and was adjusted to a flowrate at 1 mL/min and 5 µL loading volume for each sample.

The relative amount of different IgG glycans was analyzed by capillary gel electrophoresis with the LabChip^®^ GXII Touch™ HT Protein Characterization System (PerkinElmer). First, supernatant samples were purified using PreDictor MabSelect SuRe 96-well plates (Cytiva) and the Ab buffer kit (Cytiva). Buffer from protein A purification was exchanged and desalted using Vivaspin^®^ 500 Centrifugal Concentrators (Sartorius) with a molecular mass cut off at 10 kDa. Purified and desalted samples were further processed with the Glycan Release and Labeling kit (PerkinElmer) for glycan measurement with the LabChip GXII Touch (Perkin Elmer).

Values for cell specific productivity (qP) as well as integral of viable cell concentration (IVCC) determination were calculated equivalent to Janoschek et al. (2019). Integral of viable cell volume (IVCV) and specific growth rate (µ) was calculated according to Schulze et al. (2021). Product recoveries for the IH operation were calculated in correspondence to Saballus et al. (2021). All calculations are specified further in the Supplementary Eqs S1–S5.

GraphPad Prism version 9.3.1 was used for statistical evaluation given in the single graphs. Threshold for significance level was *α* = 0.05, for normal distributed data sets analysis of variance (ANOVA) with Tukey’s multiple comparison test was conducted. If standard deviation differed significantly according to Brown-Forsythe test, Dunnett’s T3 multiple comparisons test was performed.

## 3 Results

The aim of this study was to combine the advantages of a perfusion process like elongated duration and increased cell densities overtime with the low media and consumable consumption of a discontinuous process format. In detail, this new designed process, called continuous fed-batch (cFB) should comprise a fixed FB duration (FB cycle) with a consecutive rapid and complete media exchange (IH operation) including a cell density adjustment (bleed). To test the applicability of this process a high throughput small-scale screening was conducted, utilizing a commercially relevant CHO cell line, comprising different durations and starting cell counts. Subsequent, the most promising approach was scaled up to a benchtop system (5L) to investigate the influence of the scalable exchange method via the fluidized bed centrifuge (FBC).

### 3.1 Process characterization at small scale

**FIGURE 1:**
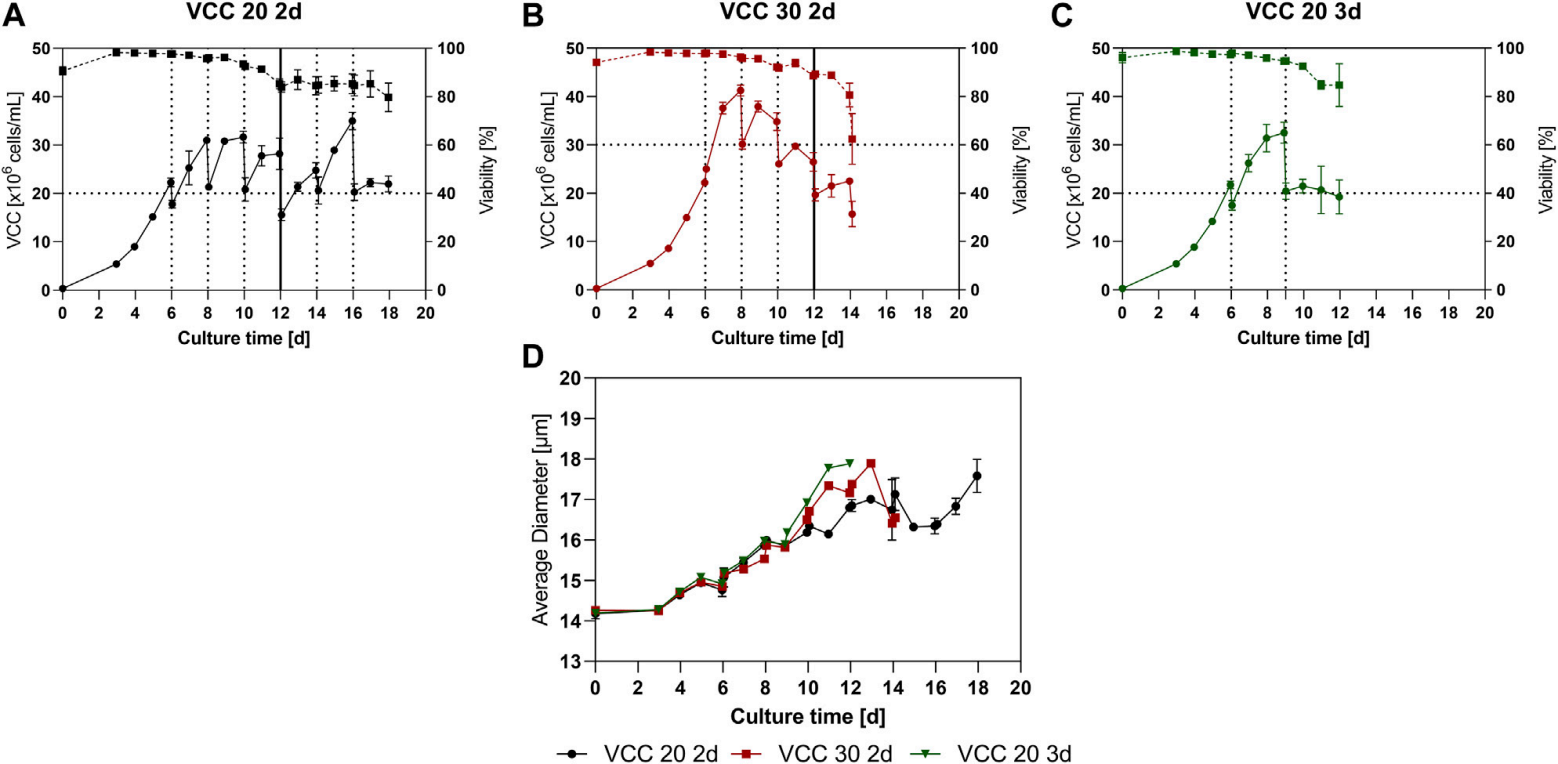
Cellular characteristics for the continuous fed-batch (cFB) process conducted in small scale. **(A–C)** Viable cell counts (solid line) and viabilities (dotted line) overtime for the scenarios VCC 20 d2 **(A)**, VCC 30 d2 **(B)** and VCC 20 d3 **(C)**. **(D)** Average diameter for each investigated approach over the course of the cultivation. All cultivations were done in duplicate and error bars represent the distance of the mean to the values obtained in both duplicates.

A small-scale trial was conducted to investigate a suitable cultivation scheme, including optimal FB cycle duration and cell density for reinoculation after the IH. Cells should remain in the logarithmic growth phase between IHs and therefore the cell bleed after each harvest cycle aims on keeping the cells in the proliferative state, defined as positive range of the cell specific growth rate (µ), to enable a continuous process mode. Three different approaches were investigated, re-inoculation at a viable cell count (VCC) of 20 × 10^6^ cells/mL and an interval of 2 days (VCC 20 2d), re-inoculation at 30 × 10^6^ cells/mL and an interval of 2 days (VCC 30 2d) and re-inoculation at 20 × 10^6^ cells/mL and an interval of 3 days (VCC 20 3d), further explanation for the design space is discussed in Section 4.1. The VCC as well as the viability for the different conditions over time is shown in Figures 1A–C. All approaches show a similar growth and viability for the first 6 days as expected and reached VCCs of around 20 × 10^6^ cells/mL. Subsequent, the first IH operations were conducted for each approach, indicated by the dotted lines.

The VCC 20 2d approach (Figure 1A) grew reproducible to a peak cell density of ∼30 × 10^6^ cells/mL over the first two FB cycles. Subsequent, from day 12 to the end of the cultivation a variation in peak cell density was visible (25–35 × 10^6^ cells/mL), partially due to variations in reinoculation densities caused by vessel change which will be discussed further in Section 4.3. This trend is supported by the specific growth rate over time visible in Supplementary Figure S1. In total six repetition could be conducted in the small-scale trials showing decreased growth in the last repetitions. The viability values for the VCC 20 d2 approach showed a decrease to 85% at day 12, after which a stabilization of viabilities is visible and a final viability of 79% is reached at day 18. Interestingly, the stabilization of the viability and high variation in peak VCC start occurring from day 12, concomitant with the change of the bioreactor vessel.

The approach with increased cell densities (VCC 30 d2) reached a peak cell density of 41.2 × 10^6^ cells/mL during the first FB phase, visible in Figure 1B. Consecutively, after the following IH operation the reinoculation cell density was set to 30 × 10^6^ cells/mL (d8) and the next FB phase was initiated. During the subsequent FB intervals, a decline in cell growth was visible, resulting in a decrease of peak cell densities for this approach, even with the bioreactor change at day 12. Similar to the VCC the viability and specific growth rate decreased over the time course, and especially after the last IH procedure at day 14 leading to the termination of the culture.

Finally, the third investigated scenario (Figure 1C) with an elongated FB duration of 3 days and reinoculation cell densities of 20 × 10^6^ cells/mL, VCC 20 3d, showed a stable cell growth after the first IH (d6) up to the end of the first FB cycle on day 9, reaching peak cell densities of 32.5 × 10^6^ cells/mL. However, after this no further cell growth, in cell count and specific growth rate, within the second FB cycle could be detected and a decrease in cell viability occurred, leading to an end of the cultivation on day 12. Further data of lactate and glucose levels can be found in the Supplementary Material.

To gain further insight into the process the average cell diameter of each approach is displayed in Figure 1D. Interestingly, all approaches show a similar increase overtime from around 14 µm up to 18 µm. Notably, both cultures that received a vessel exchange (VCC20 2d and VCC30 2d) showed a temporary decrease in cell diameter shortly after the vessel change. Afterwards, the VCC20 2d showed a stabilization of the cellular diameter up to day 17.

**FIGURE 2:**
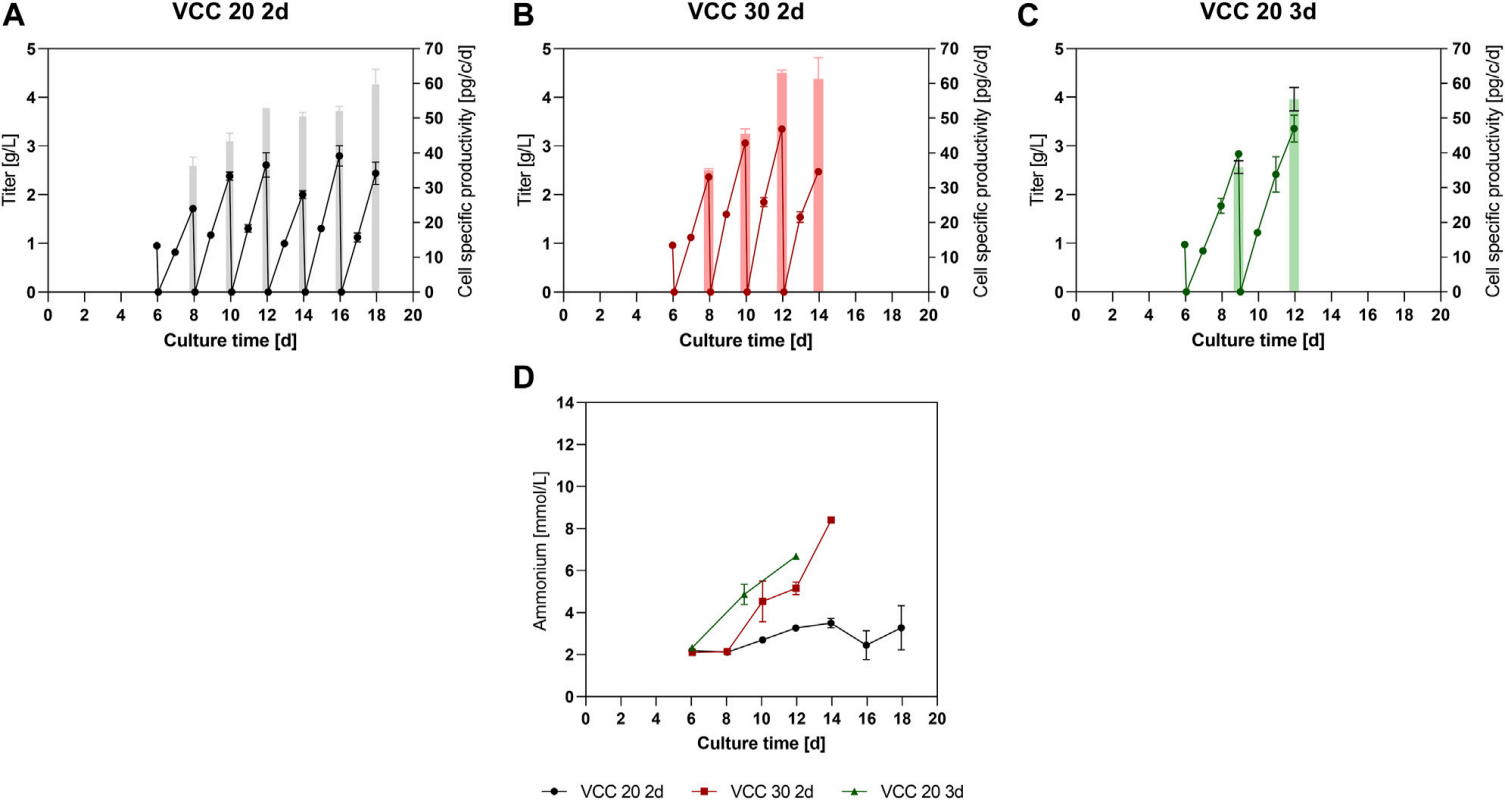
Expression levels of product and byproduct in the new designed process. **(A–C)** Titer values in g/L and cell specific productivity (pg/c/d) for the small-scale trials of the approaches VCC20 d2, VCC30 d2 and VCC 20 d3. **(D)** Ammonia level in mmol/L for each approach measured after the complete media exchange. All cultures were done in duplicate and error bars represent the distance of the mean to the values obtained in each duplicate.

To further analyze the approaches within the new designed process, product titers were quantified from day 6 on (Figures 2A–C). Thereby, all three approaches showed similar mAb titers of around 1 g/L up to day 6 before the initial IH operation. Subsequently, due to limited sampling volume at 15 mL scale no residual mAb titer sample was measured after the media exchange. Therefore, to improve visibility within Figures 2A–C, 0 g/L was assumed as starting point. For the approach VCC 20 2d, a mAb titer of 1.7 g/L (Figure 2A) could be detected after the first 2-day FB cycle on day 8. In the subsequent FB cycles, an increase to final titers of 2.0–2.8 g/L mAb, respectively, could be observed. Interestingly, an increase for the cell specific productivity (qP) over time is visible (bars in Figure 2A) from 36 pg/c/d within the first 2-day cultivation interval to 50–60 pg/c/d for the last four cycles. Accordingly, the mAb titer of the VCC 30 2d approach, Figure 2B, inclined from 2.3 g/L in the second IH (day 8) to over 3 g/L in the subsequent harvests. This increase is also reflected in the qP values, starting at around 35 pg/c/d for the second IH and reaching around 61–63 pg/c/d in the latest harvests. The titer values for the approach VCC 20 d3, shown in Figure 2C, indicate a stronger increase in titer in comparison to the VCC 20 2d approach with around 2.8–3.3 g/L in the first vs. the second 3 days of FB operation, respectively. Similar to other investigated approaches the qP values are increased from the second harvest (35 pg/c/d) to the third harvest (55 pg/c/d). Furthermore, the integrated titer over the complete cultivation duration is shown within the Supplementary Material. Interestingly, the incline in qP and titer correlates with the diameter increase shown in Figure 1D, indicating a proportional relation.

As an important byproduct and cell culture state indicator, the ammonia concentration was monitored over time. The VCC 20 d2 approach showed constant and low levels ranging between 2.2- and 3.5-mM ammonia over the entire cultivation time of 18 days. However, the ammonia concentrations for VCC 30 d2 and VCC 20 d3 increased to 6.6 and 8.4 mmol/L over the course of cultivations, indicating a significant ammonia accumulation in these cultures.

The data overall show that for each investigated approach at least 2 IH intervals could be carried out and an increase in qP with increasing process duration could be obtained, indicating a beneficial process strategy. Based on our data set the most promising approach, characterized by longest proliferating phase and low and stable amounts of ammonia over multiple IH cycles was the VCC20 d2. Therefore, the conducted small-scale screening indicates a feasible scenario for the proposed cFB process if a reinoculation cell density of 20 × 10^6^ cells/mL and an FB duration of 2 days is pursued (VCC20 d2). In accordance, this approach was chosen for a scale-up to a 5 L bioreactor scale, comprising a fluidized-bed centrifuge (FBC) as scalable cell separation method for the IH operations.

### 3.2 Proof of concept at benchtop scale

Following the process characterization of the different proposed cFB scenarios at screening scale a process scale-up with the identified preferential parameters, re-inoculation cell density 20 × 10^6^ cells/mL and a 2-day interval, was conducted. This proof-of-concept run was pursued utilizing a 5 L benchtop reactor and a single-use FBC system for time saving, gentle and complete media exchange. Two parallel cultivations were conducted, comprising one approach with enriched cell culture media, further explained in Section 2.4, as wash buffer for the FBC operation (cFBPerfM) and a second approach utilizing PBS as wash buffer (cFB PBS).

**FIGURE 3:**
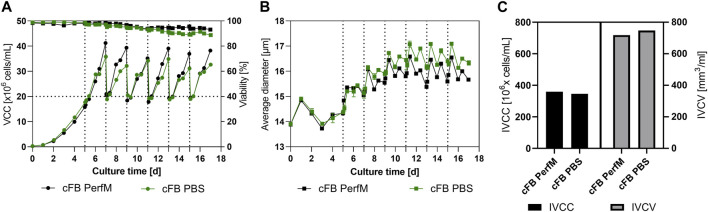
Cellular characteristics of the proof-of-Concept experiment at benchtop scale. **(A)** Viable cell counts (circles) and Viability (squares) for both experimental approaches over time, the horizontal bar shows the targeted re-inoculation VCC. **(B)** Cellular diameter for both set of experiments overtime. **(C)** Summarized Integral of viable cell count (IVCC) and Integral of viable cell volume (IVCV) for the conducted experiments. All Media exchange (IH) events were indicated by a dotted vertical line at the specific timepoints.

The course of VCC and viability for both benchtop reactors over time is displayed in Figure 3A. The cultivation conducted with enriched media as wash buffer (cFB PerfM; black line) showed overall consistent growth between the FB cycles. Overall peak cell densities after 2 days ranging from 35 × 10^6^ to 41 × 10^6^ cells/mL were reached throughout all six repetitions. This consistent growth is as well confirmed by the stable specific growth rate of around 0.37 1/d visible in the Supplementary Material. Additionally, the cell culture maintained a high cell viability of over 93% until the last day of the process. The approach utilizing PBS as wash buffer during IH operations (blue line) showed very similar trends. In this approach slightly decreased peak cell densities between 31 × 10^6^ and 35 × 10^6^ cells/mL were reached verified by a constant but lower specific growth rate of 0.29 1/d (Supplementary Material). This was accompanied by a minor reduction in viability down to 89% on day 17 in comparison to the cFB PerfM approach. Overall, cells showed better and more consisted growth behavior between IH operations as well as increased viability profiles overtime in comparison to the small-scale trials.

To further characterize the new process the average cell diameter was analyzed and is shown in Figure 3B. A similar increase for both reactors is visible during the first few days of cultivation. Subsequent, a stabilization of the diameter at 16–17 µm is visible for both set of experiments. Interestingly, after day 8 first small differences are visible with an overall increased diameter for the cFB PBS experiment of around 0.5 µm over the course of the cultivation. Overall, a specific behavior is noticeable in the diameter oscillation, with a clear increase during the first 12 h after IH operation, and a subsequent decrease over the following 12 h. Interestingly, the cell counts show an inverse behavior meaning that cells either grow in cell count or in cellular diameter within one period. To further analyze the cellular characteristics between both approaches the IVCC (Integral of Viable cell count) and IVCV (Integral of viable cell volume) were calculated. The reactor washed with PBS showed slightly decreased amounts of final IVCC for the overall process compared to the cFB PerfM (345 × 10^6^ cells/mL vs. 359 × 10^6^ cells/mL, respectively). This decrease can be explained by the small difference in peak cell densities visible in Figure 3A. Vice versa, the values for the IVCV showed a small increase for the cFB PBS experiment, as a result of the increase in diameter visible in Figure 3B.

**FIGURE 4:**
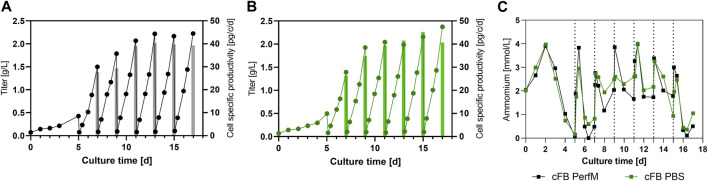
Expression characteristics for the benchtop scale reactors for the cFB experiment. **(A, B)** Titer in g/L (line) and cell specific productivity in pg/c/d (bars) for each of the conducted experiments; cFB PerfM **(A)**, cFB PBS **(B)**, respectively. **(C)** Level of Ammonia in mmol/L over the course of the cultivation for both conducted benchtop reactors.

To further gain insight into the process product specific parameters for both approaches like titer and cell specific productivity (qP) were determined and compared. Figure 3A shows the mAB titer overtime for the cFB PerfM cultivation indicated as solid line and the qP within a FB cycle represented by the individual bars. The titer curve steadily increases to the first IH operation to around 0.5 g/L, consecutively titers within FB cycles showed enhancing values from 1.5 up to 2.2 g/L in the 4th IH cycle (day 11) and then remained constant around 2.1–2.2 g/L. Similar to the titer values the qP values increased over time from around 27–40 pg/c/d at day 9 after which values remained constant around 40 pg/c/d. Similar to the results from the cFB PerfM reactor the titer in the cFB PBS reactor showed a constant increase in the first days to around 0.5 g/L. Consecutive, an increase of titer up to around 2 g/L on day 9 is visible, after which a constant value could be detected to the end of each cultivation. The qP values show similar values for the PBS reactor as for the PerfM reactor, increasing from 27 up to 40 pg/c/d, with a small outlier on day 15 with 45 pg/c/d. Additionally, during the washing step almost all mAb was removed and thus very high mAb recoveries of approximately 95% were obtained in all FBC operations (data not shown) and titer measurements after IH operation revealed only minor amounts of mAB were left behind in the cell culture resulting in low start concentrations of about 0.1 g/L at the beginning of a cycle (Figures 4A, B). Besides product titers, the ammonia levels were tracked over the term of the cultivations to monitor potential byproduct inhibition (Figure 4C). Overall, ranges between 0 and 4 mmol/L were measured, with values at harvest days ranging from 0 to 2.6 mmol/L for both cultivated reactors. These values matched with the small-scale data shown in Figure 2D for the VVC20 d2 approach. Overall, this data underlines the feasibility of the cFB approach over multiple IH and FB cycles. Furthermore, our data suggest no significant impact due to PBS washing on the process performance.

### 3.3 Process benchmark

**FIGURE 5:**
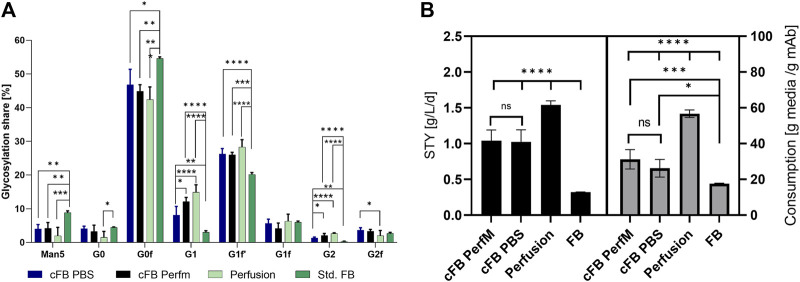
Analyzed Critical quality attributes and performance attributes for the proof of concept of the cFB cultivation in comparison to the standard FB and perfusion process. **(A)** Glycan distribution of all four conducted experiments in percentage for the eight investigated glycans. **(B)** Calculations for titer per day and reactor volume (g/L/d) and consumed dry media per mAb (g/g) for the four investigated process types.

Besides expression levels and metabolic byproducts, the glycan distribution should be investigated and compared to the standard operations. As reference a standard FB operation in duplicate 5L UniVessel, resulting in 3.85 g/L mAb in 12 days and a duplicate perfusion process in 250 mL small-scale system, with perfusion target of 50 × 10^6^ cells/mL at 2.5 VVD and 1.54 g/l/d mAb production was utilized. Both processes comprised similar cell line and media systems and are established as processes in our facility. The glycosylation pattern were investigated comparing eight different IgG glycan variants (Figure 5A). While only minor differences could be detected between both cFB variants, we were able to identify distinctions to the other process formats, except for the proportion of G1F. The majority of glycan ratios for the cFB process seemed to be rather comparable to the results of the perfusion process, such as the proportions of Man5, G1, G1F’, G0F and G2. However, the other glycan forms G0 and G2F seem to be more comparable to the FB operation. Interestingly, the data reveal a distinct difference between the continuous process types (cFB, perfusion) and the discontinuous process type (FB). However, overall the glycosylation profile of the novel process seems to be rather comparable with the perfusion glycans and exhibit a trend towards higher galactosylated forms.

In addition to the glycan distribution, performance attributes were compared to the standard process types. Therefore, the space time yield (STY) in g/L/d of the processes as well as the media consumption per produced mAb were calculated to assess benchmark characteristics in comparison to the existing process formats (Figure 5B). Of note, the STY for the continuous operations were calculated on daily basis for either the permeate (perfusion) or supernatant (cFB) within the constant phase, the discontinuous STY was calculated with the titer at the last day over the overall process time. As expected, the perfusion process showed the highest STY around 1.54 g/L/d, while the FB process only reached values of 0.32 g/L/d. Both cFB processes reached a STY in between of the standard processes around 1.0 g/L/d due to the elongated process times compared to the FB but decreased cell densities compared to the perfusion process. Furthermore, the ratio between the amount of dry media components, incorporating basal-, feed media and glucose, needed to produce 1 g of mAb was calculated as an indication of the media consumption of each process. Therefore, the total amount of vessel volume exchange per day (VVD) needed to be determined, comprising 1.3 Reactor volume per day (RV) for the cFB PerfM approach and 0.47 RV for the cFB PBS trial. Thereby, to decrease the residual PBS within the cFB PBS trials a high concentration of cells after centrifugation (+100 × 10^6^ cells/mL) were executed with the FBC system, possible throughout the functional principle of the system (Saballus et al., 2021). Thereby, this approach shows unbeneficial characteristics for the cFB PerfM trials due to increased wash buffer consumption up to 2.6 reactor volumes per single media exchange (=1.3 RV). However, former studies incorporating a different FBC method showed diminished values of 1.3 reactor volumes for a single exchange, resulting in 0.7 RV per 2 day FB cycle, which was used for calculation (Reger et al., 2023). The highest media usage per produced Gram mAb was observed for the perfusion process with ∼56 g/g, while a very low media consumption could be identified for the FB process with ∼17 g/g. Similar to the productivity, the cFB process showed a media consumption ranging in between that of both standard processes from ∼31 g/g (cFB PerfM) to ∼26 g/g (cFB PBS). As an alternative measure, the cost per mAb produced was calculated utilizing assumptions of Klutz et al., 2016, with basic medium price of 35 €/L, feed media price of 90 €/L and perfusion media price of 15 €/L. Thereby, a similar trend is visible with the lowest cost for the FB with 7.0 €/g mAb, followed by cFB PBS (12.9 €/g mAb); cFB Perf (15.4 €/g mAb) and the highest values for perfusion with 24.45 €/g mAb. This difference within the approaches is caused mainly by the optimized media exchange method in the cFB PBS trials.

Overall, the presented data proofs feasibility of the proposed FB-perfusion hybrid process over a relevant process duration of 17 days to be considered a continuous operation and successful scale-up to benchtop scale. Furthermore, the novel process allows for an efficient combination of the advantages from both standard process types, long duration and increased peak cell densities known from perfusion cultures and low media usage as in a fed batch operation. Therefore, the novel hybrid process format exhibits great potential towards highly productive continuous process.

## 4 Discussion

The aim of this study was to develop a hybrid process, that combines advantages from discontinuous process operations, such as low media consumption, with the benefits of continuous processes, such as high volumetric productivity. Therefore, a semi-continuous process operation was established, encompassing consecutive FB cycles, each started with a complete media exchange. To identify the most promising process strategy, we firstly varied essential parameters, such as inoculation VCC and FB cycle duration in a small-scale screening approach. The most promising set points could be identified as initial VCCs of 20 × 10^6^ cells/mL and a FB duration of 2 days. Subsequent, this approach was successfully transferred to a 5L bench-top reactor combined with a fluidized-bed centrifuge to enable fast, complete, and gentle media renewal. Overall, our data suggest that the established hybrid process represents a feasible and scalable new strategy to successfully combine beneficial characteristics from discontinuous and continuous process operations.

### 4.1 Small scale process development

For a first assessment of utilizable parameter set in terms of cell concentration at the start of each cycle and the duration of a cycle, the two existing process types, FB and perfusion, were used as a starting point. With the FB data a regular peak VCC of ∼25 × 10^6^ cells/mL can be reached, indicating that at this cell concentration nutrient limitations or product inhibitions started occurring for this cell line and medium, leading to a transition to the stationary phase. Meanwhile, the perfusion cultivation requires around 2.5 VVD exchange per day to keep a steady cell concentration at 50 × 10^6^ cells/mL revealing a possible media supply rate to support proliferating cells at these high densities. With this evidence we hypothesized that a single media renewal in the range of every 2–3 days should allow to sustain cell densities in the following FB cycles of 20–40 × 10^6^ cells/mL. Hereby, a reinoculation cell density of 20 × 10^6^ cells/mL and a 2-day FB duration could be identified as most promising approach, indicated by elongated cell growth over multiple cycles (six repetitions as shown in Figure 1A) and a small but acceptable decrease in cell viabilities that could partially result from biomass attaching to the vessel and building up in the bioreactors and the utilized semi-perfusion method which is further discussed in Section 4.3. Furthermore, this approach produced around 2 g/L of mAb during each interval with stable ammonia values and increasing cell specific productivities. Increased starting cell counts (30 × 10^6^ cells/mL) as well as enhanced FB cycle duration of the cFB process showed undesired process characteristics like diminished proliferation of the cells, enhanced decrease of viabilities over time and build-up of ammonia concentrations. Therefore, these processes were not subjected to further investigation due to lack in continuous process characteristics. However, this approach did show a strong increase in cell-specific productivities over time. This effect especially with increasing expression levels for the two respective cultures is a known effect from previous studies where a media exchange in the stationary phase overall increased expression, but did not result in additional cell proliferation (Reger et al., 2023). The explanation for lack of continuous process characteristics, i.e., the decreasing viabilities and cell proliferation, within these cultures could be an irreversible transition of the cells into the stationary phase without further proliferation potential. This transition can be triggered by the accumulation of metabolic byproducts, which ultimately limit further proliferation and finally reduce viabilities (Lao and Toth, 1997; Mulukutla et al., 2017; Sha et al., 2021). Another explanation for this irreversible transition of the respective cultures (30 d2; 20 d3) could be a limitation of nutrients (Xie and Wang, 1994; Xie et al., 1997; Lu et al., 2013). The commonly analyzed nutrients, such as glucose and glutamine showed no obvious limitations (data not shown), but further nutrients analysis, e.g., to assess amino acid concentrations or organic acid contents, could be conducted to investigate the nutrient levels in the unstable cultures. Nevertheless, the primary goal of this proof-of-concept study was to find a viable process set up that will allow for a continuous process operation with existing cell line and media platforms.

### 4.2 Proof of concept

In the next step the preferential process parameter set was scaled-up to two 5L bioreactor utilizing a fully scalable FBC system for cell and media separation. Both reactors were run in parallel, only differing in the media exchange method. Thereby, the reactors differed in media matrix, PBS and enriched basal media ratio. With this modification the media usage of the cell wash could be diminished to 0.47 RV instead of around 1.3 RV for a single FB cycle. Yet, due to this method small amounts (5%–10%) of PBS remained in the cell culture, which might influence process performance. Indeed, small differences between the reactors could be seen for peak cell densities, viability, and cellular diameter over time. An explanation for the decreased peak VCCs alongside the increase in cell diameter for the PBS process could be a minor shift from cell proliferation towards cell size increase. This hypothesis is supported by the IVCC and IVCV data (Figure 3C), which confirm a similar overall cell volume (IVCV) for both reactors, while slight differences in the cell counts (IVCC) can be seen. The shift from cell proliferation to cell size increase could have been triggered by the increased ion concentration that the cells of the cFB PBS reactor have been subjected to by the residual PBS. The main components of the utilized PBS are sodium, chloride and potassium ions, which are commonly utilized to induce hyper osmolality within cell cultures (Pfizenmaier et al., 2015). In accordance, these “hyper osmolality” studies have shown similar effects on cellular behavior, e.g., reduced peak cell densities and increased cell volume (Alhuthali et al., 2021; Romanova et al., 2021). Therefore, the observed shift towards cell size increase is most likely triggered by the residual PBS. However, overall our data suggest only minimal influences based on the washing buffer, since both cultivations were characterized by steady growth, high viability and comparable product titers over all six conducted repetitions (Figures 3A, B). This leads to the conclusion that PBS can be used as wash buffer without notable impact on the process, resulting it a significant reduction of nutrient-rich and costly perfusion media.

Of note, within the conducted experiment we could observe an increasing cell specific productivity for the novel process, consistent among the screening cultivations as well as the processes at bench-top scale (Figures 2A–C, 4A, B). Exemplified for the 5L cultivations, the qP values showed an increase up to 1.5-fold over the course of cultivation stabilizing around day 11 for both reactors. The increase of cell specific productivity is one of the major goals within the upstream bioprocessing to further boost the productivity with similar plant size and process operation. The enhanced cellular expression thereby is most likely connected to the respective cell diameter increase, that correlates very nicely and also reaches a steady state from ∼day 11 (Figure 3B). Further, a similar behavior could be reported in earlier studies using an FBC system (Reger et al., 2023) as well as other studies revealing a strong correlation of cellular diameter and cell specific productivity within a common FB design (Lloyd et al., 2000; Pan et al., 2017). Therefore, the new developed process format should be further analyzed to reveal the genetic and metabolic background of this beneficial behavior towards the cellular expression, but this was not the scope of this work.

Besides the expression levels of the mAb the glycosylation pattern of the product needs to be classified and compared for new designed process. Thereby the glycosylation pattern can impact the *in-vivo* half-life, therapeutic potency as well as toxicological profile of the antibody (Liu, 2015; Smith and Bertozzi, 2021). To assess these a glycan analysis was conducted release a percentage share in glycan distribution for alle conducted processes visible in Figure 5A. Thereby, significant differences were visible between the continuous and discontinuous processes for all glycans except G0, G1F and G2F. For the continuous processes shifted glycans towards higher galactosylated forms, like G1, G1F’ and G2 were visible, meanwhile Man5 and G0F showed lower shares in comparison to the discontinuous process. An explanation for the shift could be the decreased amount of inhibitory molecules for the continuous process, due to the exchange of spend media (Pereira et al., 2018). Vice versa, discontinuous process types, comprising a stationary phase, show an increased amount of inhibitory molecules. These inhibitory molecules can have an influence on intracellular processes and influence the glycosylation pattern of the product as described in different studies (Gramer and Goochee, 1993; Lipscomb et al., 2005, 2005; Rodriguez et al., 2005). One example for inhibitory molecules is ammonia, for which it has been shown in different studies to influence the galactosylation of the desired product (Borys et al., 1994; Gawlitzek et al., 2000; Yang and Butler, 2002). Interestingly, the ammonia concentration showed high discrepancy between the continuous process (≈4–6 mM) and the discontinuous operations (≈12 mM) (data not shown). Therefore, an influence of the ammonia could be suggested, but other impacts of the diverse inhibitory matrix cannot be suspended. Further studies would be needed to investigate the impact of the inhibitory matrix.

The glycans between the cFB process comprising PBS as wash media showed smaller alterations for the G1, G2 and G2F to the perfusion process. Furthermore, significant shifts between both the cFB processes for the G1 and G2 glycans were visible. An explanation for these shifts could be the increased concentration of ions, mainly sodium and chloride as main ingredients of PBS, which could impact the intracellular processes. However, this shift mainly occurs for glycan structures with low percentage share, especially for the comparison of the two IH operation, which could indicate none or just small impact of the PBS. Further studies need to be conducted to define if the changed glycans impact the expressed product. Overall, the cFB processes showed comparable glycan distributions to those of the perfusion process, while the distribution deviated from that of the discontinuous FB.

### 4.3 Process benchmark and challenges

Main goals of all process intensification strategies are to increase volumetric productivity, decrease production time or plant footprint. To show the impact of the novel process scenario a comparison with the respective standard FB as well as perfusion process was conducted. Firstly, we calculated the space time yield (STY) of all processes. We could identify increasing values from the FB process with 0.32 g/L/d over both cFB processes with 1.0 g/L/d to the perfusion process with 1.54 g/L/d (Figure 5B). The increase in STY from discontinuous to continuous processes is well known and can be mainly traced back to the significantly higher cell densities that can be maintained with continuous operations. This is in accordance with the new developed processes analyzed in this study. While the common FB process comprise a VCC from 0.3–25 × 10^6^ cells/mL, the cFB process reaches cell counts between 20–40 × 10^6^ cells/mL, while the highest cell densities could be reached with the perfusion culture at constant values around 50 × 10^6^ cells/L. A further important factor is the process duration. In this context, cultivations comprising an elongated phase with high cell densities that result in high product titers, like cFB and perfusion processes, increases the STY (MacDonald et al., 2022). Vice versa, the FB process, incorporating a substantial process time share of low cell counts during the growth phase, characterized by a lower STY value. Overall, the novel hybrid process is characterized by a significant productivity increase compared to the FB operation, represented by an impressive +217% STY, but still below (∼34%) of the values that can be reached via perfusion cultivation. However, a strong drawback of perfusion processes is the comparably high media consumption, usually exceeding the two media exchanges per day within a common perfusion cultivation (Meuwly et al., 2006; Walther et al., 2019). This could be clearly confirmed for the processes evaluated in the present study. Our data show that the respective perfusion process utilizes ∼222% more media to produce an equal amount of the mAB compared to the discontinuous FB operation (compare Figure 5B). This pattern can be easily explained, since during the last cultivation phase of the FB no cell proliferation needs to be supported by excessive media exchange, while constant proliferation and therefore nutrient supply is required for continuous processes. Interestingly, despite its continuous character, the novel cFB process shows again values between both process formats that results in a clear increase in media usage compared to FB (∼+48%) but still an up to ∼50% saving with respect to the standard continuous (perfusion) process. This low media consumption for a (semi)-continuous process can be explained by the process design which is enabled by the FBC as media renewal system. Advantages of the FBC operation are the fast and complete media exchanged at specified times. Common filter-based retention devices utilize a continuous exchange creating a intermixture of fresh and spend media in the reactor (Brantley et al., 2021). This intermixture leads to partial loss of fresh media into the following downstream operation. This loss in non-utilized media can be significantly reduced with the FBC operation due to the complete media exchange. A further advantage is the complete transfer of the produced mAB into the downstream, while during standard perfusion cultivation usually significant amounts are lost in the bleed or retained in the bioreactor due to filter sieving (Bielser et al., 2021). In contrast, due to the utilization of the FBC the complete produced mAB is harvested and fed into the subsequent downstream. Moreover, by using PBS during the wash step of the operation a 1:1 media exchange can be achieved, further decreasing the media consumption of the process.

Besides the discussed decreased media consumption and productivity, the new developed process could combine several advantageous characteristics from both cultivation types. Firstly, the elongated duration could be transferred from the continuous cultivation not only improve the STY but as well possibly diminish the number of necessary seed train runs further decreasing labor costs and good expenditures. Secondly, the cFB process comprises the process monitoring of a FB process, which is far simpler than the complex, continuous control of the peripherical devices within a filter-based perfusion cultivation. This advantage not only decrease the process monitoring but as well increase the flexibility to pursue the media exchange with the fast and gentle FBC operation. Another benefit of the utilized separation method towards filter-based system is comprised by the impossibility of filter fouling, further improve stability of the new developed process. Overall, a new process could be established comprising a continuous character with elongated plant utilization and increased productivity with the diminished media utilization and process monitoring of a discontinuous process. Further, leading to our conclusion that the continuous fed-batch shows a great potential to improve current bioprocess technologies not only in technical but as well in economical perspective.

As usual, new developments, especially within the bioprocess manufacturing, typically arise with a new set of challenges. One clear challenge is the need for suitable small-scale screening platforms (Gagliardi et al., 2019). As can be seen from the results gained in this study, repeatability of the small-scale experiments in comparison to the benchtop-scale is limited and clear difference within growth and viability at the end of the process became apparent for the scale down model, possibly caused by the utilized IH mimic method. This mimic comprised a manually exchange of media after a common dead-end centrifugation. Thereby, not only residuals of media components could be retained within the cell pellet but as well small cell debris and dead cells which subsequent could impact the performance of the cell culture within the next FB cycle of the process. These residuals of media as well as cell debris possibly can be removed by an higher extent with the FBC due to the functional principle. Therefore, a more suitable small-scale system would be desirable to increase the insight into the process on a screening level. One possibility thereby would be to develop a scale-down model for the utilized FBC system and couple it with a suitable high throughput upstream system. However, the utilized method showed adequate comparability of the current process characteristics and limitations to gain sufficient predictivity for the scale-up of the process. Very similar challenges are known for the scale down models utilized for the development of perfusion cultivation formats, were commonly semi-perfusion methods, either in spin tubes or 15 mL reactor systems, are used based on centrifugation or sedimentation strategies to renew the media. These methods proofed feasible for a first assessments of, for example, media consumption, clone feasibility and impact on CQAs (Kreye et al., 2019). However transferability of the processes into larger scale including filter membrane based cell separation methods remain limited especially in regard to continuous media flow, removal of cell debris and filter utilization (Mayrhofer et al., 2021). Nonetheless, the first processes assessments in high-throughput are important for a fast and streamlined transfer of clones and prove towards the new process strategy. Thereby, the fast and easy transfer of cell lines is one key challenge for to facilitate bioprocess development.

Further, new process strategies result in modified requirements towards the bioreactor set-up, may include new or repurposed equipment, which accordingly will require validation studies and risk assessments to be suitable for clinically relevant biopharmaceutical manufacturing processes. In the presented work, two main alterations to the platform processes and equipment have been introduced. One the one hand, an FBC system was utilized, that has been primarily been developed and used as cell harvest operation unit to separate product and cells at the end of a process. Therefore, further qualification of the FBC system for this continuous application needs to be conducted to show the feasibility especially in an GMP environment. As note, in the current study no further challenges occurred due to the continuous application, therefore we do not see any obvious obstacles from the current point of view in further qualification of this method.

Secondly, in comparison to the fed-batch and also the perfusion process the cell broth leaves the bioreactor and therefore, the controlled cultivation vessel, completely for a short period of time. However, the uncontrolled time is increased for the cFB method, in comparison to the perfusion, mainly due to the complete processing of the reactor. This elongated uncontrolled time can impact the cell culture performance and should be investigated in more depth for further risk assessments. However, for now no further negative impacts due to the uncontrolled environment could be detected in our studies. Furthermore, as mentioned, an additional step for media exchange in the cFB and perfusion is applied, therefore a similar risk or both processes can be assumed. Moreover, the increase in peripheral devices can be seen as negative impact on the process economics. Thereby, a holistic examination of the underlying process economics including all three upstream process operations and the subsequent downstream operation would be necessary to draw any comprehensive conclusion on overall economic implications of the novel process. Similar to the business case, process scale-up into pilot and production scale should be investigated within further studies. Thereby, suitable approaches like a scale-up model of the utilized FBC system are available however, a proof of concept needs to be conducted to show applicability for the mentioned scales. Despite the several challenges for implementation, the developed hybrid process operation indicates a set of significant improvements with the potential to outperform the established process operations.

## 5 Conclusion and outlook

In this study an innovative process at the interface of FB and perfusion was developed, facilitating high volumetric productivity and low media consumption in a continuous process format. Further, our proof-of concept study confirmed feasibility over extended culture durations, indicating the potential to be utilized in a continuous process approach. Due to the scalable FBC system alongside a standard FB-bioreactor this process is easy scalable and requires no physical filter-based cell retention device with the associated filter-related issues. This makes the novel process highly interesting for further investigations and process development efforts, for example, further screening of cell lines or media systems. Furthermore, transferability of the presented concept should be investigated for further processes, including non-CHO cell lines and a variety of products such as viral vectors or cell therapies. Overall, the novel developed process showed potential to further enhance the product yields by simultaneously decreasing expenditures.

## Data Availability

The raw data supporting the conclusion of this article will be made available by the authors, without undue reservation.
