# Supplementary material for: A novel hybrid bioprocess strategy addressing key challenges of advanced biomanufacturing
Source: Front Bioeng Biotechnol. 2023 Jun 30;11:1211410. doi: 10.3389/fbioe.2023.1211410 (PMC10349264; doi:10.3389/fbioe.2023.1211410)
Supplement: Supplementary file 1 [file DataSheet1.PDF]

Supplementary material

This document contain the supplementary data for the Manuscript “A Novel Hybrid Bioprocess Strategy Addressing Key Challenges of Advanced Biomanufacturing” by Lucas Nik Reger, Martin Saballus, Annika Kappes, Markus Kampmann, Rene H. Wijffels, Dirk E. Martens, Julia Niemann.

Firstly, all utilized calculation with descriptions of the single variables are stated.

The Integral of Viable cell count (IVCC) were calculated over time (t) by trapezoidal integration of the VCC. The calculation is shown in Equation 1

$$IVCC_i = \int_{t=0}^{t} VCC(t)dt \approx \sum_{i=1}^{n} \frac{VCC_i + VCC_{i-1}}{2} \times (t_i - t_{i-1})$$

Equation 1: Calculation for integral of viable cell count (IVCC).

For calculation of the viable cell volume firstly the daily specific cell volume (VCV) needs to be determined by equation 2 including the cellular diameter (d_{cell}). Subsequent the similar calculation for IVCC determination was utilized (equation 3).

$$VCV = \frac{4}{3} \times \pi \times VCC \times \left(\frac{d_{cell}}{2}\right)^3$$

Equation 2: Calculation for the specific volume (VCV)

$$IVCV_i = \int_{t=0}^{t} VCV(t)dt \approx \sum_{i=1}^{n} \frac{VCV_i + VCV_{i-1}}{2} \times (t_i - t_{i-1})$$

Equation 3: Integral of viable cell volume (IVCV) over the course of cultivation

The cell specific productivity () was calculated by the equation 4 including the daily mAb titer (c) and the IVCC (c_{Cell}) between the specific timepoints (t).

$$qP = \frac{c_i + c_{i-1}}{t_i - t_{i-1}} \times \left(\frac{VCC_i + VCC_{i-1}}{t_i - t_{i-1}}\right)^{-1}$$

Equation 4: Calculation of the cell specific productivity (qP) over the course of cultivation.

The specific growth rate (µ) was determined to the following equation.

$$\mu = \frac{\ln\left(\frac{VCC_i}{VCC_{i-1}}\right)}{t_i - t_{i-1}}$$

Equation 5: Calculation for the specific grwoth rate (µ) of the cultivation

To calculate the mAb recovery (*R*) of the fluidized-bed-centrifuge (FBC), the mass of mAb in the FBC harvest (m_H) and the mass of mAb in FBC waste (m_W) were taken into account.

$$R = \frac{m_H}{m_H + m_W} \times 100$$

Equation 6: Calculation for the mAb recovery rates for the fluidized-bed-centrifuge operations.

To further support the understanding of the current work extra cellular or metabolic characteristics were attached to this file. Thereby, firstly the al supplementary data for the small scale cultivation is state. In Figure 1 the specific growth rate for the small-scale trials stated over the course of cultivation for all approaches are shown.

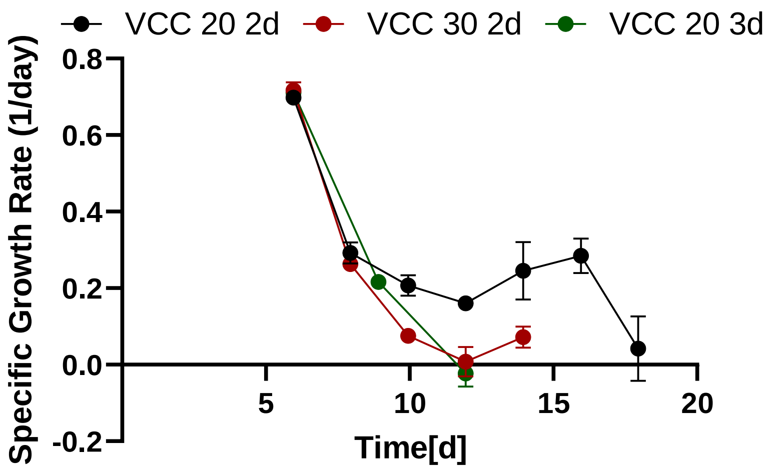

Figure 1: Specific growth rate in (1/day) for all small scale cultivations.

Figure 2 shows the glucose values for the small-scale cultivation over the course of cultivation.

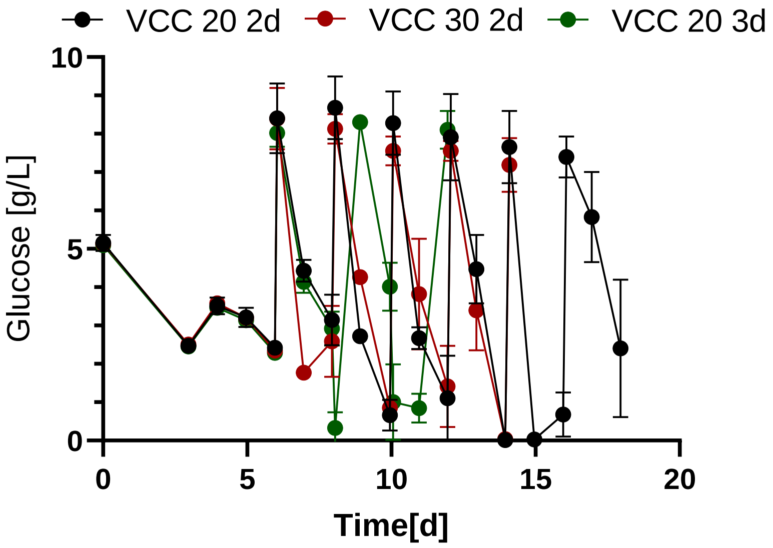

Figure 2: Glucose levels in g/L over for the small-scale cultivation.

Lactate values for the small-scale cultivation are shown in Figure 3.

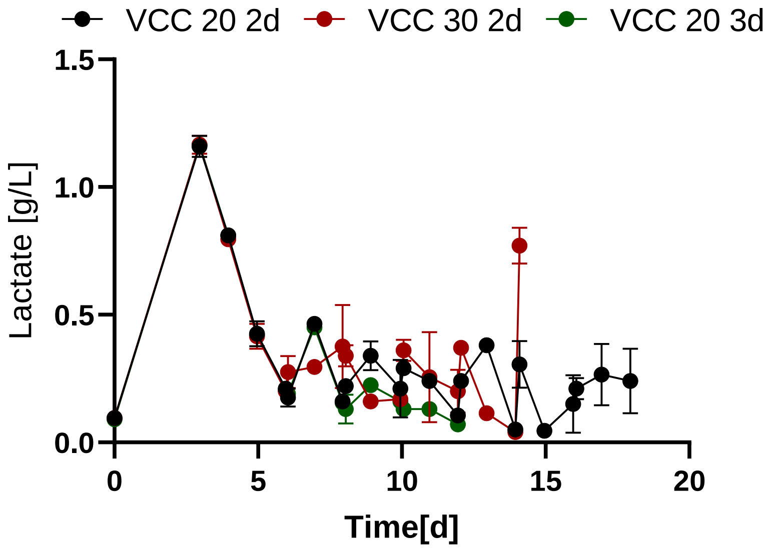

Figure 3:Lactate [g/L] for all small scale cultivations for the cultivated time period.

The cumulated titer within the small scale cultivation are shown in Figure 4

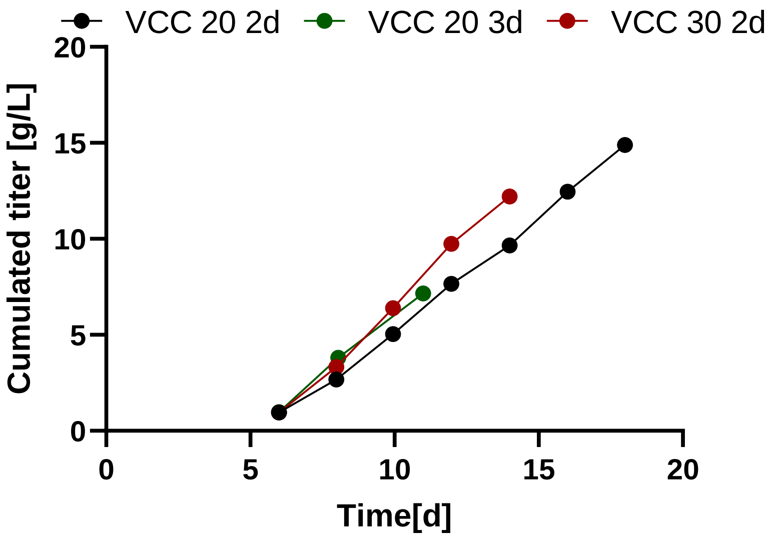

Figure 4: Cumulated titer for the cultivations within the screening system in g/L.

Specific growth rate for the proof of concept cultivation within the two UV 5L reactors are shown in Figure 5

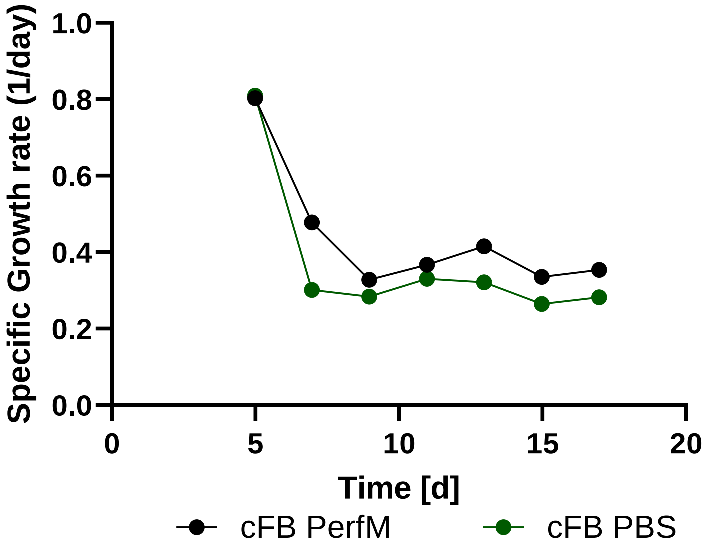

Figure 5: Specific growth rate for the PoC cultivation in 5L scale.

Figure 6 reveals the glucose values for the PoC cultivation in benchtop-scale.

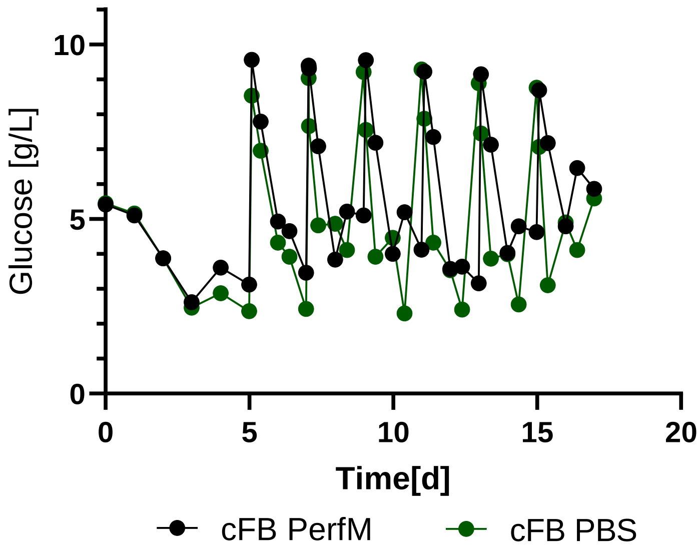

Figure 6: Gluoce [g/L] for the PoC in benchtop scale.

Figure 7 shows the lactate values over the course of cultivation.

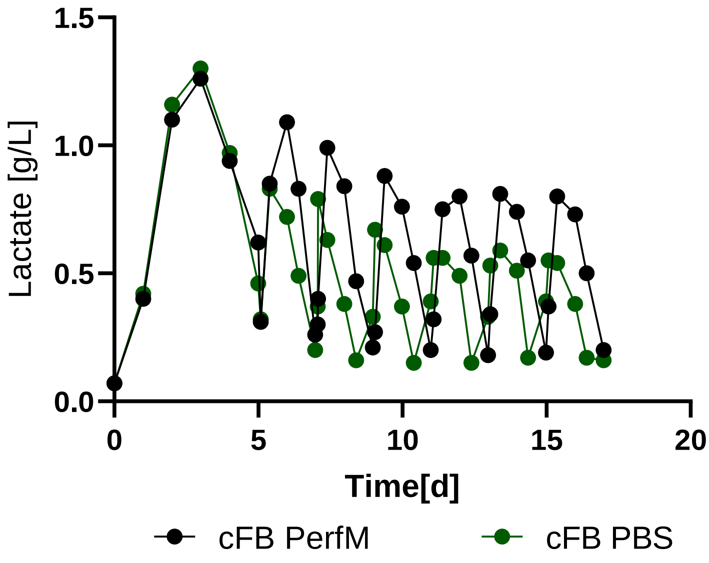

Figure 7: Lactate values for the 5L scale cultivation.

Glutamine levels in mM for both conducted benchtop cultivation are visible in Figure 8.

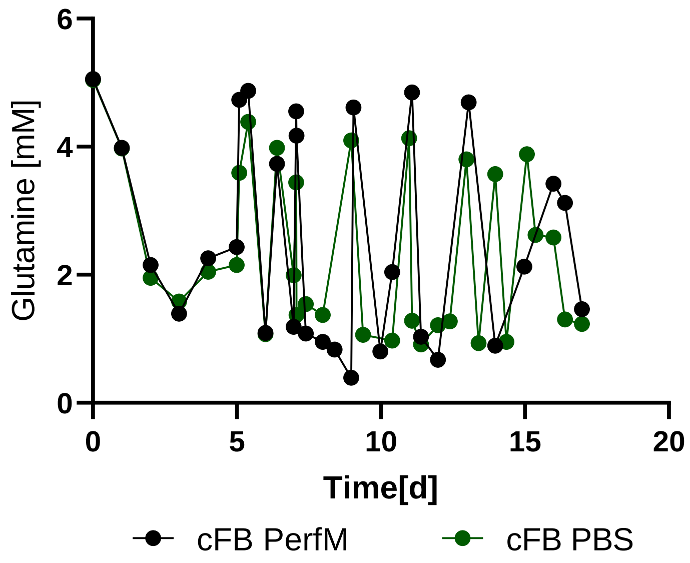

Figure 8: Glutamine concentrations in mM over the cultiation duration for both 5L scale PoC reactors.

The cumulated titer for the UV 5L cultivation is shown in Figure 8

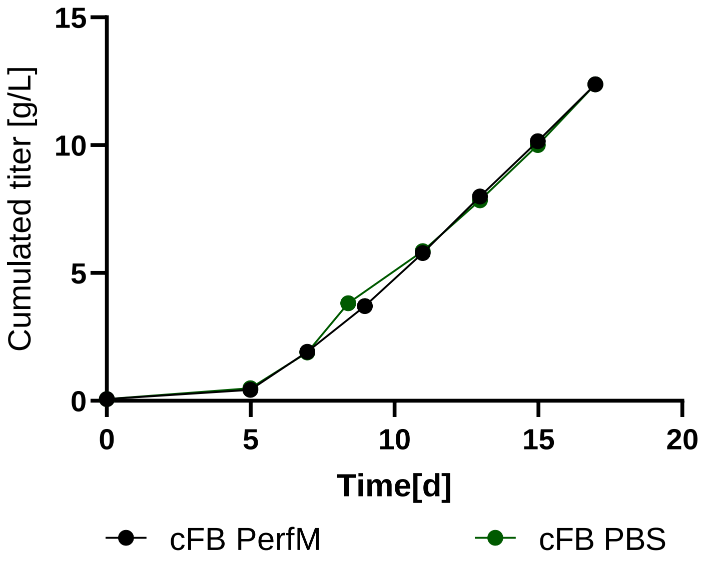

Figure 9: Cumulated titer over the course of cultivation for the 5L UV.

Besides further cultivation parameter of the new hybrid process cFB, a summary of the two standard processes were attached to this file. Figure 10 shows the standard fed-batch cultivation of the utilized clone. The axis allocation is signaled by the arrows.

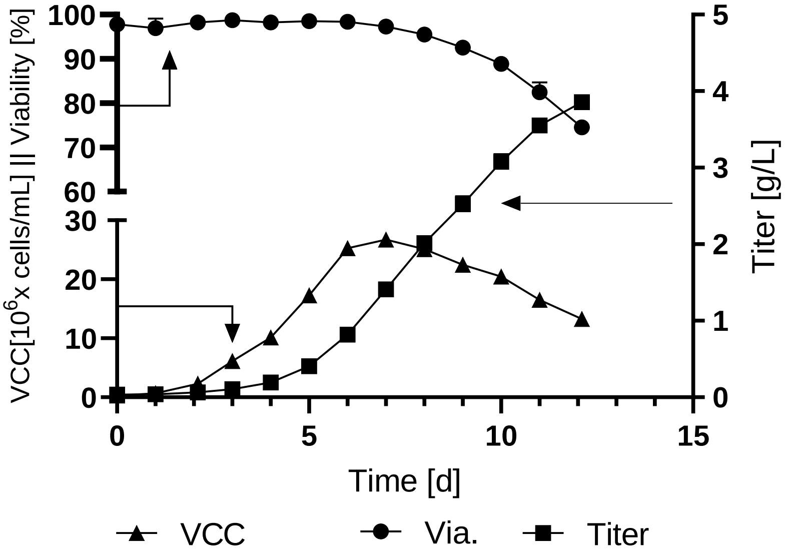

Figure 10: Standard Fed-batch cultivation of the utilized clone within a 5 UniVessel in a duplicate cultivation.

Furthermore, the perfusion the standard perfusion cultivation within an ambr250 system is shown in Figure 11.

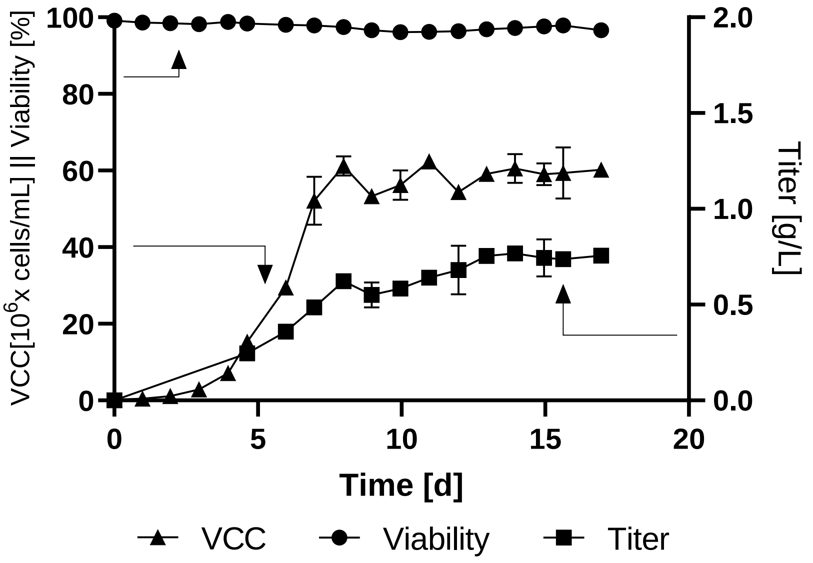

Figure 11: Perfusion cultivation with an ATF based filter system in an ambr250 system. The cultivation was runned in duplicate with goal of 50x106 cells/mL at 2.5 volume exchange per day.
